# Integrated experimental and computational insights into the anti-inflammatory potential of flower-derived exosome-like nanoparticles targeting the NF-κB pathway

**DOI:** 10.3389/fbinf.2026.1737325

**Published:** 2026-02-16

**Authors:** Sreyoshi Routh, Venkatraman Manickam

**Affiliations:** School of Bio Sciences and Technology, Vellore Institute of Technology, Vellore, Tamil Nadu, India

**Keywords:** docking, exosome-like nanoparticles, inflammation, molecular dynamics simulation, NF-κB

## Abstract

**Introduction:**

Dysregulated inflammation underlies numerous chronic pathologies, with the NF-κB p65–p50 heterodimer acting as a pivotal transcriptional regulator that mediates different inflammatory responses. Consequently, inhibiting NF-κB nuclear translocation has emerged as a promising strategy in anti-inflammatory drug development. While floral extracts have been widely used, recent advances have highlighted the therapeutic potential of flower-derived exosome like nanoparticles as promising cell-free therapeutics owing to their enhanced biocompatibility and stability.

**Methods:**

Exosome like nanoparticles were isolated from three ethnomedicinal flowers and systematically characterized. Antioxidant potential of ELNs was evaluated through DPPH assay and their anti-inflammatory potential was assessed. Further, to elucidate the molecular mechanisms underlying NF-κB modulation, key ELN-associated metabolites were computationally screened against the crystallized NF-κB p65–p50 heterodimer using molecular docking, followed by molecular dynamics simulations to evaluate binding stability and interaction dynamics.

**Results and discussion:**

Isolated ELNs demonstrated a strong antioxidant potential and *in vitro* analysis revealed significant regulation in mRNA expression of inflammatory cytokines and NF-κB transcriptional activity. Molecular docking identified several metabolites with higher binding affinity against NF-κB p65–p50 heterodimer supported by simulation studies confirming stable ligand-protein interaction. Both docking scores and simulation trajectories strongly supported stable, high-affinity interactions consistent with NF-κB pathway inhibition. Overall, the combined experimental and computational findings in the study represent the first comprehensive data of floral ELN bioactives, offering the significant translational potential of floral nanovesicles as a new class of biocompatible, cell-free nanotherapeutics for anti-inflammatory drug discovery.

## 1 Introduction

Inflammation is our body’s natural response against any kind of external stimuli; it involves various immune cells and molecular mediators to restore homeostasis (Chen et al., 2018). While acute inflammation is protective to the body, chronic inflammation is implicated in the onset and progression of a wide range of diseases such as cardiovascular, gastrointestinal, and neurodegenerative diseases and cancer (Castanheira and Kubes, 2019). A central mediator of this process is the nuclear factor kappa-light-chain-enhancer of activated B cells (NF-κB) pathway, particularly the p65–p50 heterodimer, which drives the expression of key pro-inflammatory cytokines such as TNF-α, IL-1β, and IL-6 (Biswas and Bagchi, 2016). Consequently, strategies aimed at inhibiting NF-κB nuclear translocation are considered promising for anti-inflammatory therapy.

Traditionally, flowers have been valued for their aesthetic and aromatic properties, with several species also being employed in complementary therapies such as aromatherapy and flower-based medicine due to their rich phytochemical profiles which include flavonoids, polyphenols, terpenoids, and alkaloids (Mahire and Patel, 2024). Among these, *Chrysanthemum morifolium*, *Agave amica*, and *Nerium oleander* hold particular ethnomedicinal relevance in southern India, where they are commonly used in cultural rituals such as deity ordination, worship, and traditional healing practices for inflammatory ailments (Singhal and Das, 2012; Mohamad and Che Zahari, 2024; Nahar Rahmatullah et al., 2019).

Recent advances in nanobiotechnology have uncovered a novel class of natural nanocarriers—plant-derived exosome-like nanoparticles (ELNs)—which are significantly revolutionizing plant-based therapeutics. These nanoscale vesicles, typically ranging 30 to 200 nm in diameter, are secreted by various plant tissues and are structurally similar to mammalian exosomes (Kalluri and LeBleu, 2020). Functionally, ELNs serve as intercellular communication vehicles capable of delivering encapsulated biomolecules, including small RNAs (miRNAs), proteins, lipids, and metabolites, to recipient cells across species barriers, thereby mediating cross-kingdom signaling (Boukouris and Mathivanan, 2015; Koh et al., 2023; Jiyah et al., 2024). Compared to crude plant extracts, ELNs offer several therapeutic advantages: they exhibit improved biocompatibility, are naturally encapsulated in lipid bilayers that protect their encapsulated cargo from enzymatic degradation, and possess enhanced stability in the gastrointestinal tract, making them suitable for oral delivery (Zhang et al., 2022; Khosravi et al., 2022). Moreover, ELNs have demonstrated immunomodulatory potential, particularly in inflammatory models, by modulating macrophage polarization, regulating cytokine expression, and interacting with key signaling pathways such as NF-κB (Kantarcıoğlu et al., 2023; Ou et al., 2023). While ELNs have been extensively studied from various edible fruits and vegetables (You et al., 2021; Liu et al., 2022; Sundaram et al., 2022; Yıldırım et al., 2024; Ju et al., 2013; Iriawati et al., 2024; jun et al., 2021; Perut et al., 2021), there is a relative paucity of data regarding ELNs derived from floral sources (Chen et al., 2022; Wang et al., 2024). Given that flowers are rich in secondary metabolites with established pharmacological properties, their ELNs may represent a more efficient and targeted way of delivering these bio-actives. Hence, we seek to understand the therapeutic potential and molecular composition of three different ethnomedicinal floral ELNs which has not been uncovered.

The present study focuses on ELNs isolated from *C. morifolium*, *A. amica*, and *N. oleander*, which are three culturally and medicinally significant flowers traditionally used in India. These flowers were selected based on their well-documented phytochemical composition, especially their crude extracts that have been widely studied as possessing therapeutic properties (Singhal and Das, 2012; Mohamad and Che Zahari, 2024; Nahar Rahmatullah et al., 2019). However, the application of their secreted ELNs as nano-formulated, cell-free therapeutic agents is yet to be systematically investigated. Therefore, the current study aims to bridge this gap by characterizing the phytochemical composition of ELNs derived from these flowers using gas chromatography–mass spectrometry (GC-MS) and evaluating their therapeutic potential via *in vitro* and *in silico* analyses. To investigate the modulatory action of the flower-derived ELNs on NF-κB signaling, initial *in vitro* studies were conducted, including cytotoxicity profiling, ELISA, and gene expression studies. Based on the *in vitro* findings, the identified ELN metabolites from GC-MS analysis were further screened for drug-likeliness and toxicity prediction and subjected to molecular docking against the NF-κB p65–p50 heterodimer (PDB ID: 1VKX), followed by molecular dynamics simulations to assess their binding stability, interaction profiles, and conformational dynamics.

To the best of our knowledge, this study represents the first comprehensive investigation into the molecular constituents of ELNs from these floral species and their potential role in modulating inflammatory signaling. By integrating the emerging concept of plant-derived nanovesicles with modern computational tools, our findings offer a novel perspective on the therapeutic utility of floral ELNs and lay the groundwork for their incorporation into future plant-based nanomedicine strategies.

## 2 Materials and methods

### 2.1 Collection of flowers and isolation of flower-derived ELNs using ultracentrifugation

Flowers from *A. amica*, *N. oleander*, and *C. morifolium* were acquired from Vellore district, Tamil Nadu, India. They flowers were authenticated by Dr. D. Stephen, Assistant Professor in the Department of Botany, American College, Madurai, Tamil Nadu, India. The flower petals were carefully removed and washed with distilled water to remove any impurities. In brief, 60 gm of fresh flower petals were correctly ground with 1× PBS at 1:2 ratio using a mixer grinder. The flower mixture was filtered through Whatman filter paper (pore size: 11 μm), then subjected to a series of centrifugations: 3,000 × *g* for 10 min, 6,000 × g for 20 min, and 10,000 × g for 30 min to eliminate larger cellular debris. The supernatant was carefully collected in a fresh tube, filtered through a 0.22-µm syringe filter, and subjected to ultracentrifugation for 2 h at 1,20,000 × g in 4 °C. The pellet obtained after ultracentrifugation was further washed with 1× PBS and again ultracentrifuged at 1,20,000 × g for 2 h. The supernatant was removed; the pellet was dissolved in ice-cold 1× PBS and stored at −80 °C for further experiments.

### 2.2 Characterization of flower-derived ELNs

Following the isolation of the three different flower-derived ELNs of *N. oleander* (NELNs), *C. morifolium* (CELNs), and *A. amica* (AELNs), their size, distribution, and concentration were characterized by nanoparticle tracking analysis (NTA) using a NanoSight NS300 system. In addition, to further assess the colloidal stability of the isolated ELNs, zeta potential analysis was performed using a Particle Metrix ZetaView analyzer. Furthermore, FE-SEM (fluorescent emission scanning electron microscopy) was used to morphologically characterize floral ELNs. To visualize the ELNs through FE-SEM, floral ELNs were diluted ten-fold, and 100 μL of the sample was aliquoted on a cover slip. The ELNs were fixed using 2.5% glutaraldehyde, washed thrice with 1× PBS, and further dehydrated using 30%, 50%, 70%, 80%, 90%, and 100% ethanol. The samples were finally subjected to FE-SEM (Model: FEI Quanta 250 FEG, Thermo Fisher Scientific) for morphological characterization.

### 2.3 Metabolite profiling of flower-derived ELNs using GC-MS analysis

To characterize the bioactive constituents present in flower-derived ELNs, GC-MS analysis was employed for comprehensive metabolite profiling. For phytochemical screening, the floral ELN pellet was dissolved in 5 mL of absolute methanol until homogeneity in the solution was achieved (Iriawati et al., 2024), and the metabolome was further processed for GC-MS analysis. This analysis for the flower-derived ELNs was performed in TUV-SUD, Ranipet, India using an Agilent system with a DB-5 MS capillary column (30 m × 250 µm × 0.1 µm). Initially, the oven temperature was maintained at 50 °C and later increased to 150 °C until reaching the final temperature of 250 °C. As a carrier, helium gas was inserted at a pressure of 7.5 psi, and 1 µL of sample aliquot was injected into the column. Total mass spectra were obtained at 70 eV with sections ranging from 45 to 450 Da, with a scan interval of 0.5 s. The MassHunter software platform was used to analyze the mass spectra and chromatograms, and the relative percentage quantity of each component was determined by comparing its average peak area to the total areas. Metabolites retrieved at different retention times were represented as individual mass spectra and identified using the NIST (National Institute of Standards and Technology) database (Chikowe et al., 2025). From the hit list obtained, a score threshold of minimum 50% was considered on the basis of confidence level while selecting the metabolites (Chikowe et al., 2025).

### 2.4 Antioxidant assay for the flower-derived ELNs

The protocol for DPPH (2,2-diphenyl-1-picrylhydrazyl) assay was adopted following Iriawati et al. (2024). In brief, 0.2 mM of DPPH reagent was prepared with 100 mL of absolute methanol and incubated in the dark for 2 h. The ELN metabolites were purified using methanol, and 900 µL of 0.2 mM DPPH was added to 100 µL of ELN metabolites and further incubated in the dark for 30 min at room temperature. Absorbance was measured at 517 nm using a microplate reader (BioTrek, United States). Absolute methanol was used as blank, and only DPPH reagent was used as a control. The percentage of radical scavenging activity was measured using the equation “% radical scavenging = {(AC–AS)/AC} × 100,” where AC denotes absorbance of control and AS denotes absorbance of sample. Ascorbic acid was used as a positive control.

### 2.5 Cell viability assay for flower-derived ELNs

An MTT (3-(4,5-dimethylthiazol-2-yl)-2,5-diphenyltetrazolium bromide) assay was performed to evaluate the effect of flower-derived ELNs on the cell viability of RAW264.7 macrophages, which were maintained using DMEM (Dulbecco’s modified Eagle medium) supplemented with 10% FBS (fetal bovine serum) and 5% antibiotic–antimycotic solution. In brief, 5 × 10^3^ cells were seeded in a 96-well plate and incubated in a humidified incubator at 37 °C, 5% CO_2_ for 24 h. The cells were then treated with the different flower-derived ELNs—NELNs, CELNs, and AELNs—at varying concentrations ranging from 1 to 10 μg/mL overnight. MTT (5 mg/mL) was added to the cells post treatment and incubated for 4 h, followed by the addition of DMSO (100 μL) to assess the cells’ viability (Zhai et al., 2025).

### 2.6 Preparation of nuclear extract and binding activity of NF-κB

Following the instructions provided by the nuclear extraction kit’s manufacturer (Cayman Chemicals, India), nuclear extracts were prepared from control and treated RAW264.7 macrophages. To further prevent protein degradation, the cells were quickly homogenized in phosphatase-inhibitor-containing ice-cold PBS. The homogenates were centrifuged at 300 × *g* for 5 min. Thence, the pellets were again suspended in the hypotonic buffer and detergent. The cytoplasmic fractions were then created by centrifuging at 14,000 × g for 30 s. The nuclear proteins were dissolved in lysis solution containing proteasome inhibitors after the cell nuclei had been lysed by the nuclear extraction buffer. Following the manufacturer’s instructions, the NF-κB (p65) transcription factor assay ELISA kit (Cayman) was used to assess the NF-κB DNA binding activity in the nuclear extract after being prepared.

### 2.7 Gene expression analysis using real-time PCR

In brief, LPS-induced RAW cells were treated with flower-derived ELNs and indomethacin as a positive control for 24 h. Post treatment, mRNA was isolated from the cells using the TRIzol method, and the mRNA was quantified using a NanoDrop (Eppendorf). This mRNA was further converted to cDNA using a PrimeScript™ RT reagent kit, and the cDNA was further amplified using SYBR Premix Ex TaqTMII (Tli RNase Plus) against specific forward and reverse primers (listed in Table 1) following the kit protocol. We used GAPDH as the internal standard, and the results were expressed in terms of fold change compared to control.

**TABLE 1:** Forward and reverse primer sequences for three genes.

Gene	Forward sequence	Reverse sequence	Accession number
*GAPDH*	5′-CATCACTGCCACCCAGAAGACTG-3′	5′-ATGCCAGTGAGCTTCCCGTTCAG-3′	NM_008084
*TNF-α*	5′-GGTGCCTATGTCTCAGCCTCTT-3′	5′-GCCATAGAACTGATGAGAGGGAG-3′	NM_013693
*IL-10*	5′-TACCACTTCACAAGTCGGAGGC-3′	5′-CTGCAAGTGCATCATCGTTGTTC-3′	NM_010548

### 2.8 Statistical analysis

For *in vitro* validation, we used GraphPad Prism 8.0 for statistical analysis. The data were represented as mean ± SD, n = 3 (three technical replicates), and three independent biological replicates. Moreover, one-way ANOVA with Dunnett’s or Tukey’s multiple comparisons as required was utilized to analyze the group differences, with P-value <0.05 being statistically significant.

### 2.9 Computational prediction of ligand drugability and toxicity

The metabolites identified from flower-derived ELNs were evaluated for their drug-likeliness property and toxicity profile using ProTox II. Drug-likeliness property can be demonstrated using the Lipinski rule of five, where the molecular weight of the ligand should be ≤500 kDa, H-bond donors ≤5, H-bond acceptor ≤10, and C log P (octanol-water partition coefficient) ≤5. The canonical smiles for each of the metabolites were inserted into ProTox II and the compounds were screened for favoring the Lipinski rule of five.

### 2.10 Interaction of the flower-derived ELN metabolites and NF-κB p65–p50 heterodimer through molecular docking studies

To understand the interaction of the flower-derived ELN metabolites with NF-κB p65–p50 heterodimer, the metabolites identified from GC-MS analysis were further subjected to molecular docking against NF-κB p65–p50 heterodimer (PDB id: 1VKX) to assess their binding affinities with 1VKX.

#### 2.10.1 Protein preparation

**FIGURE 1:**
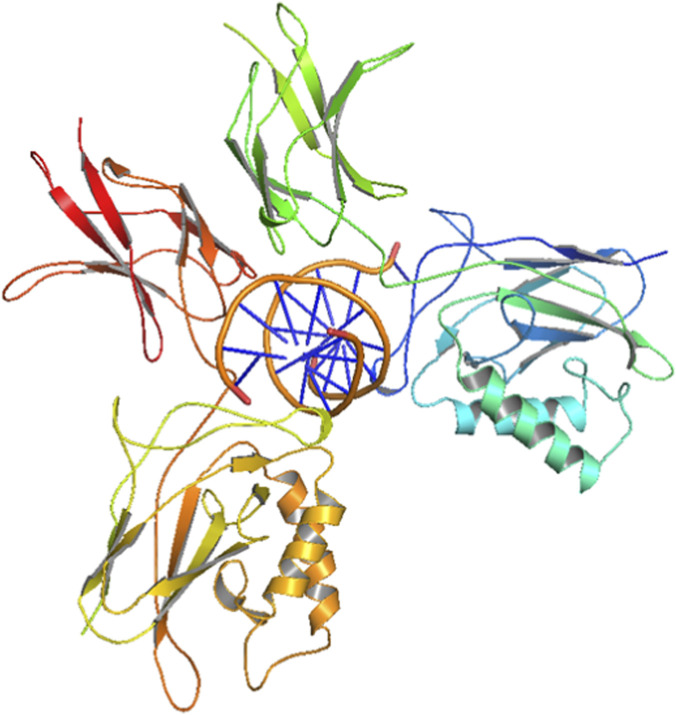
Three-dimensional structure of NF-κB p50 homodimer (PDB id: 1NFK).

For the present study, the protein of interest, NF-κB p65–p50 heterodimer, was retrieved from the RCSB Protein Data Bank with PDB id: 1VKX. The protein structure consisted of p65 and p50 subunits (chain A and B) with a DNA duplex bound to it (Figure 1). The protein was prepared using PyMOL by removing the existing co-crystallized DNA macromolecule and water molecule. In addition, hydrogen atoms and Kollman charges were assigned to the protein. This structure was later used as the preliminary structure for further docking studies.

#### 2.10.2 Ligand retrieval and preparation

The different metabolites encapsulated in the floral ELNs were identified from GC-MS data and subsequently evaluated for their potential binding affinities with 1VKX. We retrieved 3D structures for 61 compounds from PubChem and SpectraBase, and the structures were saved in structure data file (SDF) format. The ligands were prepared using OpenBabel GUI version 2.4.1 software for energy minimization and conversion of SDF file to PDBQT format (Subramani and Venugopal, 2025). Molecular docking was performed following ligand and target preparation.

#### 2.10.3 Molecular docking studies

Molecular docking studies for the selected phytocompounds derived from the ELNs of the three different flowers and the target protein 1VKX was achieved using AutoDock Vina software (Shanthi, 2022). Additionally, dexamethasone was used as a reference drug for molecular docking. The binding site of the target protein was selected based on the DNA binding region in 1VKX, and the grid box was generated with grid center coordinates X = −0.678, Y = 28.657, and Z = 54.530, comprising grid spacing of 1 Å and point spacing of 60 × 60 × 60 (Dammalli et al., 2024). The binding energies were calculated, and the 2D/3D interactions were visualized for the compounds showing the highest binding affinity of each flower-derived ELN using Discovery Studio and PyMOL.

### 2.11 Molecular dynamics simulation

The ligand–protein interactions and binding stability of the selected docking poses were further investigated through classical all-atom molecular dynamics (MD) simulations (Vishwakarma and Mittal, 2024) with the help of GROMACS software. Initial coordinates for the simulations were obtained from the docking-derived conformations. The protonation states of protein residues were assigned at physiological pH 7.0, and hydrogen atoms were added accordingly. Initially, the topology files for proteins and ligands were prepared using the CHARMM27 all-atom force field and SwissParam web server. Each protein–ligand complex was solvated in a cubic box containing water molecules using the TIP3P model and neutralized with appropriate counter ions (Jorgensen et al., 1983; Jyoti et al., 2025). Energy was minimized to 10 kJ/mol. Following initial energy minimization, the system was subjected to a 500 ps NVT simulation with the protein atoms constrained, allowing relaxation of the solvent environment, followed by a 500 ps NPT equilibration to relax the protein atoms. Subsequently, 200 ns production MD simulations were performed under an NPT ensemble. The coordinates for every MD trajectory file were recorded for every 10 ps. Following this, parameters such as RMSD (root mean square deviation), RMSF (root mean square fluctuation), SASA (solvent-accessible surface area), Rg (radius of gyration), and H-bond were analyzed using XM-grace software.

### 2.12 Prediction of binding free energies

Binding free energies were computed using MMPBSA (molecular mechanics Poisson–Boltzmann surface area) following MD simulation (Subramani and Venugopal, 2025). This software calculates the binding free energy between receptor and ligand. In this study, the final 10 ns were utilized to estimate the binding energies between the complexes, which were extracted from a 200 ns trajectory. The below approach was used for calculating the binding free energies, where ΔE_MM_ represents total molecular mechanics energy, ΔS denotes conformational entropy contribution (-TΔS), ΔG_PB_ represents polar contribution, and ΔG_SA_ refers to non-polar contribution.

$$\Delta G_{\text{bind}}=G_{\text{complex}}-G_{\text{protein}}-G_{\text{ligand}}=\Delta H+\Delta G_{\text{solvation}}-T\Delta S=\Delta E_{\text{MM}}+\Delta G_{\text{PB}}+\Delta G_{\text{SA}}-T\Delta S.$$

## 3 Results

### 3.1 Isolation and characterization of flower-derived ELNs

**FIGURE 2:**
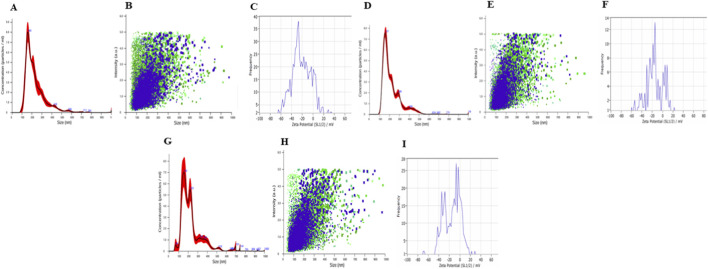
Particle size concentration, distribution, and zeta potential of flower-derived ELNs. **(A–C)** *Nerium oleander*-derived ELNs (NELNs); **(D–F)** *Chrysanthemum morifolium*-derived ELNs (CELNs); **(G–I)** *Agave amica*-derived ELNs (AELNs).

**FIGURE 3:**
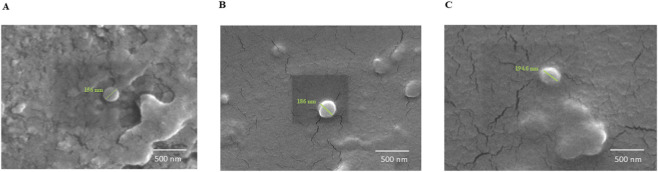
Morphological characterization of isolated ELNs using FE-SEM: **(A)** NELNs, **(B)** CELNs, and **(C)** AELNs.

ELNs were isolated using ultracentrifugation, then purified and resuspended in 1× PBS. These ELNs were also analyzed using NTA for size distribution and particle concentration. The NTA results confirmed that most of the isolated ELNs ranged 100–200 nm in diameter, and the particle concentration was 9 × 10^9^ particles/mL (NELNs), 8.5 × 10^9^ particles/mL (CELNs), and 9.7 × 10^9^ particles/mL (AELNs) (Figure 2). The average zeta potential of the isolated ELNs was found to range from −40 mV to −10 mV (Figure 2). The particle sizes of the ELNs were in accordance with previously evaluated morphological characterization of ELNs, and the negative zeta potential exhibited better stability for all the flower-derived ELNs; hence, we proceeded with further studies. For morphological assessment, isolated floral ELNs were visualized using FE-SEM (Figure 3). The ELNs exhibited vesicular morphology, with dimensions of 100–200 nm—consistent with our NTA findings.

### 3.2 Identification of metabolites from flower-derived ELNs using GC-MS analysis

**FIGURE 4:**
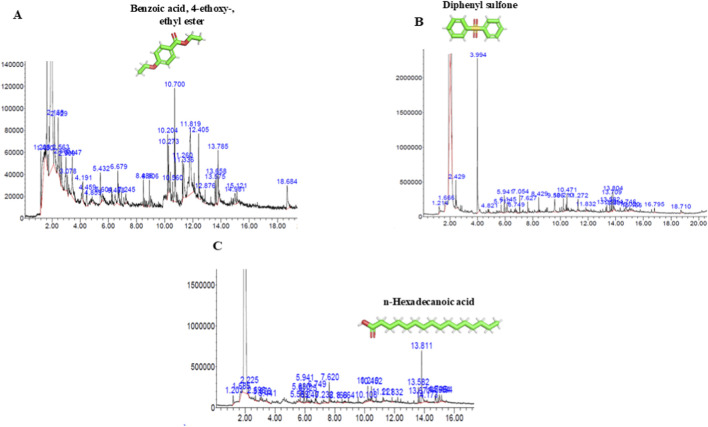
GC-MS chromatogram for metabolites derived from floral ELNs depicting the most abundant compound. X-axis represents retention time; Y-axis represents compound abundance. **(A)** NELNs **(B)** CELN, and **(C)** AELN metabolites.

Metabolome extraction was carried out from three different flower-derived ELNs using methanol following the protocol of Iriawati et al. (2024); the metabolite profiling was carried out using Agilent GC-MS system. A total of 33, 26, and 28 peaks were observed in ELNs derived from *N. oleander*, *C. morifolium*, and *A. amica*, respectively. Each peak designated the presence of metabolites relating to their molecular formula, retention time (RT), and molecular weight referred from NIST (Figure 4). However, based on a confidence level of at least 50% (Chikowe et al., 2025), the compounds were selected and documented with their peak area (%) and retention time in Tables 2–4. Most of the metabolites identified in the flower-derived ELNs have diverse protective roles such as antimicrobial, antioxidant, anti-inflammatory, anticancer and neuroprotective effects. For instance, the presence of compounds such as acetamide, 2-(adamantan-1-yl)-N-(1-adamantan-1-ylethyl), phenol, 2,5-bis(1,1-dimethylethyl)-, and n-hexadecanoic acid identified in *N. oleander*-derived ELNs are known to possess anticancer, antimicrobial, and antioxidant properties. On the other hand, the presence of 2-Caren-4-ol in *C. morifolium*-derived ELNs has antioxidant and antimicrobial properties, and hexadecanoic acid present in *A. amica*-derived ELNs is known to have a potent anti-inflammatory role.

**TABLE 2:** Metabolites identified from *Nerium oleander*-derived ELNs (NELNs) through GC-MS analysis.

S. no.	Compound	Molecular formula	Molecular weight (g/mol)	Retention time (min)	Peak area (%)
1	Silanediol, dimethyl	C_2_H_8_O_2_Si	92.17	2.665	1.22
2	2-Oxopropanoic acid	C_3_H_4_O_3_	88.06	2.989	1.75
3	3-Amino-2-oxazolidinone	C_3_H_6_N_2_O_2_	102.09	3.447	2.56
4	m-xylene and p-xylene	C_8_H_10_	106.16	4.191	0.81
5	Phenol	C_6_H_6_O	94.11	5.432	1.29
6	1,3-Cyclopentanedione, 2,4-dimethyl	C_7_H_10_O_2_	126.15	6.488	0.82
7	Undecane	C_11_H_2_4	156.31	6.679	1.63
8	2-Propyl-tetrahydropyran-3-ol	C_8_H_16_O_2_	144.21	7.245	0.42
9	Pentacosane	C_25_H_52_	352.7	8.486	0.5
10	Octadecane	C_18_H_38_	254.5	8.906	0.74
11	Cyclopropane, nonyl	C_12_H_24_	168.32	10.204	1.27
12	Phenol, 2,5-bis(1,1-dimethylethyl)-	C_14_H_22_O	206.32	10.56	0.42
13	Benzoic acid, 4-ethoxy-, ethyl ester	C_11_H_14_O_3_	194.23	10.7	3.89
14	Diethyl phthalate	C_12_H_14_O_4_	222.24	11.26	0.77
15	1,3,4,5-Tetrahydroxycyclohexanecarboxylic acid	C_7_H_12_O_6_	192.17	11.336	2.23
16	cTetradecanoic acid	C_14_H_28_O_2_	228.37	12.405	1.98
17	Acetamide, 2-(adamantan-1-yl)-N-(1-adamantan-1-ylethyl)	C_24_H_37_NO	355.6	12.876	0.5
18	Hexadecanoic acid, methyl ester	C_17_H_34_O_2_	270.5	13.575	0.63
19	7,9-Di-tert-butyl-1-oxaspiro(4,5)deca-6,9-diene-2,8-dione	C_17_H_24_O_3_	276.4	13.658	0.57
20	n-Hexadecanoic acid	C_16_H_32_O_2_	256.42	13.785	1.34
21	5-Nitro-2,4(1H,3H)-pyrimidinedione	C_4_H_3_N_3_O_4_	157.08	14.981	0.74
22	7H-3-[5-Methyl-1-(4-methylphenyl)-1,2,3-triazol-4-yl]-1,2,3-triazol-4-yl]-6-(6-methoxynaphthalen-2-yl)-s-triazolo [3	C_23_H_17_N_7_S	423.5	18.684	1.78

**TABLE 3:** Metabolites identified from *Chrysanthemum morifolium*-derived ELNs (CELNs) through GC-MS analysis.

S. no.	Compound name	Molecular formula	Molecular weight (g/mol)	Retention time (mins)	Peak area (%)
1	Dimethyl sulfone	C_2_H_6_O_2_S	94.14	3.994	8.64
2	Ethanol, 2-(2-ethoxyethoxy)-	C_6_H_14_O_3_	134.17	5.712	0.39
3	1-Hexanol, 2-ethyl-	C_8_H_18_O	130.229	5.941	0.75
4	2-Pyrrolidinone, 1-methyl-	C_5_H_9_NO	99.13	6.145	0.66
5	Nonanal	C_9_H_18_O	142.24	6.749	0.68
6	Bicyclo [3.1.1]hept-2-en-6-one, 2,7,7-trimethyl-	C_10_H_14_O	150.22	7.054	0.75
7	Ethanol, 1-(2-butoxyethoxy)-	C_8_H_18_O_3_	162.23	7.627	0.6
8	3,6-Octadienoic acid, 3,7-dimethyl-, methyl ester, (Z)-	C_11_H_18_O_2_	182.26	8.429	1.09
9	1,3-Cyclohexadiene-1-carboxaldehyde, 2,6,6-trimethyl	C_10_H_14_O	150.22	9.586	1.7
10	2-Tetradecene, (E)-	C_14_H_28_	196.37	10.210	0.84
11	Germacrene D	C_15_H_24_	204.35	10.417	0.7
12	Diethyl phthalate	C_12_H_14_O_4_	222.24	11.272	0.71
13	1-Hexadecanol	C_16_H_34_O	242.44	11.832	0.52
14	2-Caren-4-ol	C_10_H_16_O	152.23	13.353	0.39
15	Hexadecanoic acid, methyl ester	C_17_H_34_O	270.5	13.582	0.48
16	3,4,5-Trimethylpyrazole	C_6_H_10_N_2_	110.16	13.709	0.83
17	n-Hexadecanoic acid	C_16_H_32_O_2_	256.42	13.804	1.08
18	Benzothiazole, 2-(2-hydroxyethylthio)-	C_9_H_9_NOS_2_	211.3	13.881	0.45
19	Methyl hexadec-9-enoate	C_17_H_32_O_2_	268.4	14.746	0.83
20	Benzenamine, 4,4′-methylenebis-	C_13_H_14_N_2_	310.5	15.166	0.5
21	2-Methyl-5H-dibenz [b,f]azepine	C_15_H_13_N	207.27	18.710	0.52

**TABLE 4:** Metabolites identified from *Agave amica*-derived ELNs (AELNs) through GC-MS analysis.

S. no.	Compound	Molecular formula	Molecular weight (g/mol)	Retention time (mins)	Peak area (%)
1	1-[2,5-Dimethoxy-4-(methylsulfonyl)phenyl]-2-propanamine	C_12_H_19_NO_4_S	273.35	1.201	0.48
2	Ethanol, 2-(2-ethoxyethoxy)-	C_6_H_14_O_3_	134.17	5.68	0.34
3	1-Hexanol, 2-ethyl	C_8_H_18_O	130.227	5.941	0.51
4	2-Pyrrolidinone, 1-methyl	C_5_H_9_NO	99.13	6.125	0.35
5	Nonane	C_9_H_2_0	128.25	6.24	0.16
6	Nonanal	C_9_H_18_O	142.24	6.749	0.81
7	2,3-Dihydro-3,5-dihydroxy-6-methyl-4H-pyran-4-one	C_6_H_8_O_4_	144.12	7.232	0.48
8	Ethanol, 1-(2-butoxyethoxy)-	C_8_H_18_O_3_	162.23	7.62	0.56
9	N-[3-[(6-Keto-9-nitro-5H-benzo[b][1,4]benzoxazepin-7-yl)oxy]phenyl]acetamide	C_21_H_15_N_3_O_6_	405.37	8.664	0.24
10	Cyclopropane, nonyl	C_12_H_24_	168.32	10.21	0.5
11	Benzene, 1,2-dimethoxy-4-propenyl-, (Z)-	C_11_H_14_O_2_	178.23	10.452	0.67
12	1-Hexadecanol	C_16_H_34_O	242.44	11.832	0.16
13	Hexadecanoic acid, methyl ester	C_17_H_34_O_2_	270.5	13.582	0.47
14	2H-2a,7-Methanoazuleno [5,6-b]oxirene, octahydro-3,6,6,7a-tetramethyl	C_15_H_24_O	220.35	13.671	0.23
15	n-Hexadecanoic acid	C_16_H_32_O_2_	256.42	13.811	2.3
16	cis-13-Octadecenoic acid, methyl ester	C_19_H_36_O_2_	296.5	14.746	0.64
17	9-Octadecenoic acid	C_18_H_34_O_2_	282.5	14.994	0.48
18	Octadecanoic acid	C_18_H_36_O_2_	284.5	15.134	0.37

### 3.3 Quantitative analysis of free radical scavenging capacity of ELNs using DPPH assay

**FIGURE 5:**
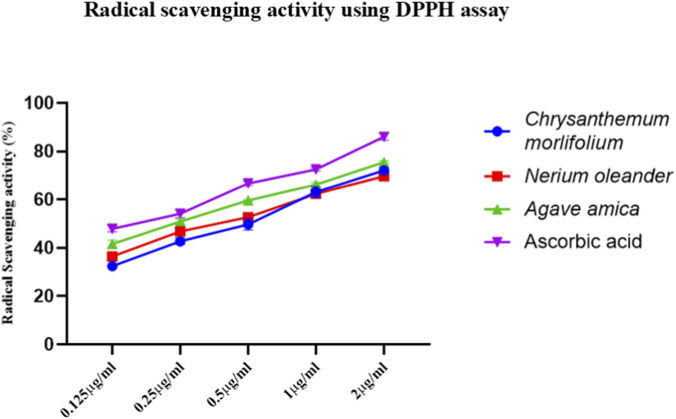
Radical scavenging activity of flower-derived ELNs using DPPH assay and ascorbic acid as standard. Data presented as mean ± SD (n = 3).

A DPPH assay was performed to evaluate the antioxidant capacity of flower-derived ELNs at different concentrations, with ascorbic acid as a positive control. ELNs were observed to exhibit radical scavenging activity in a dose-dependent manner compared to the positive control (ascorbic acid) (Figure 5).

### 3.4 Effect of NELNs, CELNs, and AELNs on the cell viability of RAW264.7 macrophages

**FIGURE 6:**
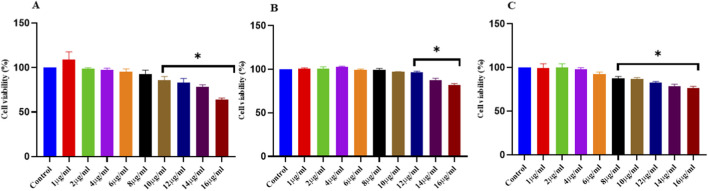
Cell viability of **(A)** NELNs, **(B)** CELNs, and **(C)** AELNs on RAW264.7 macrophages in a dose-dependent manner. Data presented as mean ± SD (n = 6), *P < 0.05 versus control.

RAW264.7 macrophages were seeded and treated with NELNs, CELNs, and AELNs at different concentrations of 1–16 μg/mL for 24 h. The medium was then removed, and MTT (5 mg/mL) was used on the treated cells to check their viability. No toxicity was observed in NELN, CELN, and AELN doses ranging 1–8 μg/mL, 1–10 μg/mL, and 1–6 μg/mL, respectively. However, higher concentrations of all three types of flower-derived ELNs resulted in a marked reduction in cell viability (Figure 6). Based on the cell viability assay, the least cytotoxic concentrations of each ELN type were selected for subsequent experimental analyses.

### 3.5 Effect of flower-derived ELNs on NF-κB binding activity, mRNA expression levels of pro-inflammatory cytokine (TNF-α), and anti-inflammatory cytokine (IL-10)

**FIGURE 7:**
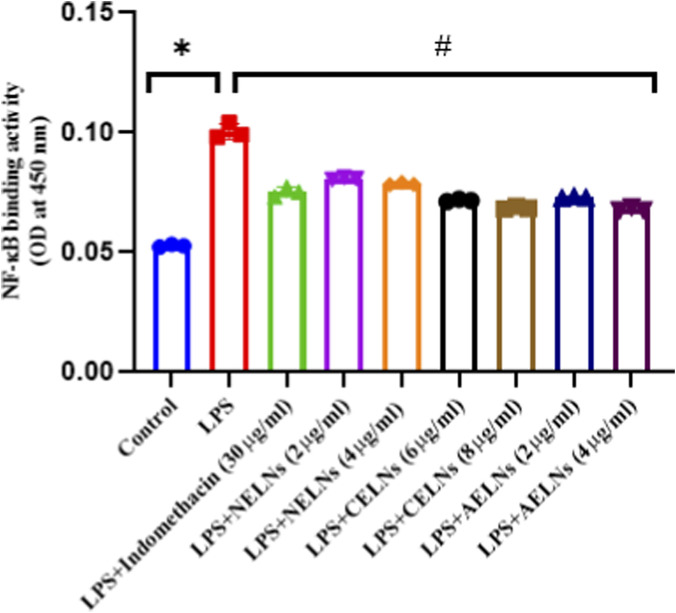
Effect of NELNs, CELNs, and AELNs on NF-κB binding activity in LPS-induced RAW264.7 macrophages. Data presented as mean ± SD (n = 3); *P < 0.05 versus control and ^#^P < 0.05 versus LPS.

**FIGURE 8:**
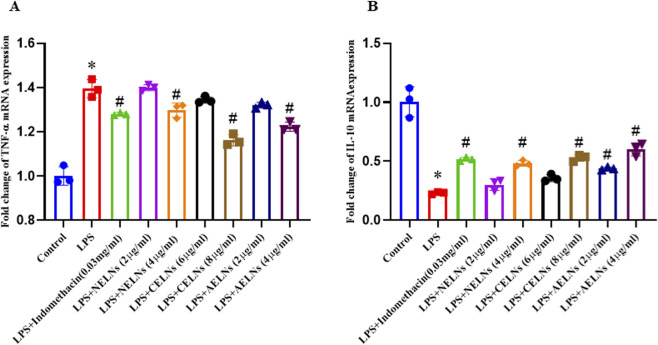
Effect of NELNs, CELNs, and AELNs on mRNA expression levels of **(A)** pro-inflammatory (TNF-α) and **(B)** anti-inflammatory (IL-10) cytokines in LPS-induced RAW macrophages. mRNA expression normalized with GAPDH as the housekeeping gene. Data expressed as fold change compared to control and presented as n = 3, mean ± SD, *P < 0.05 versus control, and ^#^P < 0.05 versus LPS.

To validate the anti-inflammatory potential of the flower-derived ELNs, LPS-induced RAW macrophages (1 μg/mL for 16 h) were treated with NELNs (2 μg/mL and 4 μg/mL), CELNs (4 μg/mL and 6 μg/mL), TELNs (2 μg/mL and 4 μg/mL), and indomethacin (positive control) (30 μg/mL) for 24 h. NF-κB binding activity was detected using ELISA, and it was significantly elevated in the LPS-treated condition, whereas flower-derived ELN-treated groups significantly reduced the condition compared to the control drug (Figure 7). Gene expression studies revealed an increase in IL-10 expression and decrease in TNF-α expression (expressed as fold change ±SD), confirming the anti-inflammatory role of NELNs, CELNs, and AELNs (Figures 8A,B) compared to positive control. TNF-α mRNA expression was significantly elevated during LPS simulation, increasing from 1 ± 0.05-fold in the untreated group to 1.41 ± 0.06-fold, while treatment with NELNs, CELNs, and AELNs significantly reduced the fold change increase to ∼1.25, ∼1.29, and ∼1.25, respectively. There was also a significant decrease in IL-10 mRNA expression, dropping from 1 ± 0.05 in untreated group to 0.20 ± 0.03 following LPS treatment. However, the same was restored upon treatment with floral ELNs, leading to an increase in fold change (NELNs: ∼0.6, CELNs: ∼0.6 and AELNs: ∼0.725) compared to LPS treatment. Notably, the higher dosage of flower-derived ELNs efficiently resulted in a significant increase of TNF-α expression and increase in IL-10 expression compared to the lower dosage for all the floral ELNs. Treatment with indomethacin as reference drug used at a dosage of 30 μg/mL showed significant reduction of NF-κB binding activity, TNF-α mRNA expression level (fold change: 1.29 ± 0.05), and a significant elevation of IL-10 mRNA expression (fold change: 0.45 ± 0.04) compared to the LPS-treated group in RAW macrophages.

### 3.6 Drug-likeliness and toxicity prediction of the flower-derived ELN metabolites

The phytocompounds identified from the GC-MS analysis of ELNs derived from three different flowers were further evaluated for their drug-likeness properties. Ligand assessment was performed based on Lipinski’s rule of five, with the results summarized in Tables 5–7. Toxicity profiles were predicted using the ProTox-II server, highlighting the estimated LD_50_ (median lethal dose), toxicity class, and organ-specific toxicities such as hepatotoxicity, neurotoxicity, and nephrotoxicity. As presented in Tables 5–7, most of the metabolites were consistent with Lipinski’s rule of five, indicating their favorable drug-likeness. Additionally, most compounds exhibited LD_50_ values ranging from 500 to 10,000 mg/kg, corresponding to toxicity classes 3–6. Organ toxicity predictions revealed that most compounds were non-hepatotoxic, non-neurotoxic, and non-nephrotoxic in nature, suggesting a generally favorable safety profile. Based on the drug-likeness and toxicity assessments, the majority of the phytocompounds derived from flower-based ELNs demonstrated favorable pharmacological properties and a low predicted toxicity profile, highlighting that ELNs might serve as superior drug candidates with enhanced safety and efficacy profiles.

**TABLE 5:** Drug-likeliness and toxicity prediction for metabolites of NELNs.

Compound	Molecular weight (g/mol)	H-bond donor	H-bond acceptor	LogP	Hepatotoxicity	Neurotoxicity	Nephrotoxicity	Predicted LD_50_ (mg/kg)	Predicted toxicity class
2-Oxopropanoic acid	88.06	1	3	−0.34	Inactive	Inactive	Active	200	3
Silanediol, dimethyl	92.17	2	3	−0.33	Inactive	Inactive	Inactive	3,160	5
3-Amino-2-oxazolidinone	102.09	1	4	−0.05	Inactive	Active	Active	1,500	4
m-Xylene and p-xylene	106.16	0	0	2.3	Inactive	Active	Inactive	2,119	5
Phenol	94.11	1	1	1.39	Inactive	Active	Inactive	270	3
1,3-Cyclopentanedione, 2,4-dimethyl	126.15	0	2	0.8	Inactive	Inactive	Inactive	2,600	5
Undecane	156.31	0	0	4.54	Inactive	Inactive	Inactive	750	3
2-Propyl-tetrahydropyran-3-ol	144.21	1	2	1.33	Inactive	Inactive	Inactive	3,100	5
Pentacosane	352.7	0	0	10	Inactive	Inactive	Inactive	750	3
Octadecane	254.5	0	0	7.27	Inactive	Inactive	Inactive	750	3
Cyclopropane, nonyl	168.32	0	0	4.54	Inactive	Inactive	Inactive	880	3
Phenol, 2,5-bis(1,1-dimethylethyl)-	206.32	1	1	3.99	Inactive	Inactive	Inactive	1,000	4
Benzoic acid, 4-ethoxy-, ethyl ester	194.23	0	3	2.26	Inactive	Inactive	Active	2,040	5
Diethyl phthalate	222.24	0	4	2.04	Inactive	Inactive	Active	6,172	6
1,3,4,5-Tetrahydroxycyclohexanecarboxylic acid	192.17	5	6	−2.32	Inactive	Inactive	Active	9,800	6
cTetradecanoic acid	228.37	1	2	4.77	Inactive	Inactive	Inactive	900	4
Acetamide, 2-(adamantan-1-yl)-N-(1-adamantan-1-ylethyl)	355.6	1	2	5.7	Inactive	Active	Inactive	3,200	5
Hexadecanoic acid, methyl ester	270.5	0	2	5.64	Inactive	Inactive	Inactive	5,000	5
7,9-Di-tert-butyl-1-oxaspiro (4,5)deca-6,9-diene-2,8-dione	276.4	0	3	3.59	Inactive	Inactive	Inactive	900	4
n-Hexadecanoic acid	256.42	1	2	5.55	Inactive	Inactive	Inactive	900	4
5-Nitro-2,4(1H,3H)-pyrimidinedione	157.08	2	3	−0.51	Inactive	Inactive	Inactive	1,923	4
7H-3-[5-Methyl-1-(4-methylphenyl)-1,2,3-triazol-4-yl]-1,2,3-triazol-4-yl]-6-(6-methoxynaphthalen-2-yl)-s-triazolo [3	423.5	0	7	4.1	Inactive	Active	Inactive	1,000	4

**TABLE 6:** Drug-likeliness and toxicity prediction for metabolites of CELNs.

Compound	Molecular weight (g/mol)	H-bond donor	H-bond acceptor	LogP	Hepatotoxicity	Neurotoxicity	Nephrotoxicity	Predicted LD_50_ (mg/kg)	Predicted toxicity class
Dimethyl sulfone	94.13	0	2	0.74	Inactive	Inactive	Inactive	5000	5
Ethanol, 2-(2-ethoxyethoxy)-	134.17	1	3	0.03	Inactive	Inactive	Inactive	4970	5
1-Hexanol, 2-ethyl-	130.23	1	1	2.2	Inactive	Inactive	Inactive	1000	4
2-Pyrrolidinone, 1-methyl-	99.13	0	2	0.18	Inactive	Active	Inactive	3914	5
Nonanal	142.24	0	1	2.94	Inactive	Inactive	Inactive	5000	5
Bicyclo [3.1.1]hept-2-en-6-one, 2,7,7-trimethyl-	150.22	0	1	2.18	Inactive	Active	Inactive	5000	5
Ethanol, 1-(2-butoxyethoxy)-	162.23	1	3	1.16	Inactive	Inactive	Inactive	2650	5
3,6-Octadienoic acid, 3,7-dimethyl-, methyl ester, (Z)-	182.26	0	2	2.85	Inactive	Inactive	Inactive	5000	5
1,3-Cyclohexadiene-1-carboxaldehyde, 2,6,6-trimethyl	165.23	1	2	2.75	Inactive	Inactive	Inactive	2500	5
2-Tetradecene, (E)-	196.37	0	0	5.48	Inactive	Inactive	Inactive	5000	5
Germacrene D	204.35	0	0	4.89	Inactive	Inactive	Inactive	5300	5
Diethyl phthalate	222.24	0	4	2.04	Inactive	Inactive	Active	6172	6
1-Hexadecanol	242.44	1	1	5.46	Inactive	Inactive	Inactive	1000	4
2-Caren-4-ol	152.23	1	1	1.97	Inactive	Active	Inactive	2340	5
Hexadecanoic acid, methyl ester	270.5	0	2	5.64	Inactive	Inactive	Inactive	5000	5
3,4,5-Trimethylpyrazole	110.16	1	1	1.33	Active	Inactive	Inactive	375	4
n-Hexadecanoic acid	256.42	1	2	5.55	Inactive	Inactive	Inactive	900	4
Benzothiazole, 2-(2-hydroxyethylthio)-	211.3	1	3	2.38	Active	Inactive	Inactive	1017	4
Methyl hexadec-9-enoate	268.44	0	2	5.42	Inactive	Inactive	Inactive	3000	5
Benzenamine, 4,4′-methylenebis-	310.48	2	2	5.84	Inactive	Active	Inactive	950	4
2-Methyl-5H-dibenz [b,f]azepine	207.27	1	1	4.36	Inactive	Active	Inactive	350	4

**TABLE 7:** Drug-likeliness and toxicity prediction for metabolites of AELNs.

Compound	Molecular weight (g/mol)	H-bond donor	H-bond acceptor	LogP	Hepatotoxicity	Neurotoxicity	Nephrotoxicity	Predicted LD_50_ (mg/kg)	Predicted toxicity class
1-[2,5-Dimethoxy-4-(methylsulfonyl)phenyl]-2-propanamine	273.35	1	5	2.78	Inactive	Inactive	Inactive	1410	4
Ethanol, 2-(2-ethoxyethoxy)-	134.17	1	3	0.03	Inactive	Inactive	Inactive	4970	5
1-Hexanol, 2-ethyl	130.23	1	1	2.2	Inactive	Inactive	Inactive	1000	4
2-Pyrrolidinone, 1-methyl	99.13	0	2	0.18	Inactive	Active	Inactive	3914	5
Nonane	128.26	0	0	3.76	Inactive	Inactive	Inactive	750	3
Nonanal	142.24	0	1	2.94	Inactive	Inactive	Inactive	5000	5
2,3-Dihydro-3,5-dihydroxy-6-methyl-4H-pyran-4-one	144.13	2	4	−0.26	Inactive	Inactive	Active	595	4
Ethanol, 1-(2-butoxyethoxy)-	162.23	1	3	1.16	Inactive	Inactive	Inactive	2650	5
N-[3-[(6-Keto-9-nitro-5H-benzo[b][1,4]benzoxazepin-7-yl)oxy]phenyl]acetamide	405.36	2	7	5.44	Active	Inactive	Inactive	1600	4
Cyclopropane, nonyl	168.32	0	0	4.54	Inactive	Inactive	Inactive	880	3
Benzene, 1,2-dimethoxy-4-propenyl-, (Z)-	178.23	0	2	2.74	Inactive	Active	Inactive	2500	5
1-Hexadecanol	242.44	1	1	5.46	Inactive	Inactive	Inactive	1000	4
Hexadecanoic acid, methyl ester	270.5	0	2	5.64	Inactive	Inactive	Inactive	5000	5
2H-2a,7-Methanoazuleno [5,6-b]oxirene, octahydro-3,6,6,7a-tetramethyl	220.35	0	1	3.63	Inactive	Inactive	Inactive	5000	5
n-Hexadecanoic acid	256.42	1	2	5.55	Inactive	Inactive	Inactive	900	4
cis-13-Octadecenoic acid, methyl ester	296.49	0	2	6.2	Inactive	Inactive	Inactive	3000	5
9-Octadecenoic acid	282.46	1	2	6.11	Inactive	Inactive	Inactive	48	2
Octadecanoic acid	284.48	1	2	6.33	Inactive	Inactive	Inactive	900	4

### 3.7 Molecular docking assessment revealed the interaction profiling of ELN metabolites with NF-κB p65–p50 heterodimer

**FIGURE 9:**
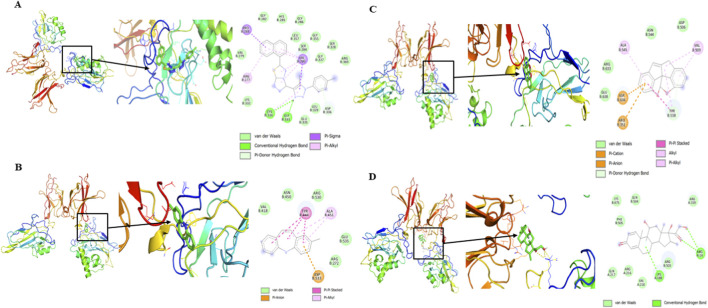
Two- and three-dimensional visualizations of top three ligand protein interactions from the three different flower-derived ELNs. **(A)** 7H-3-[5-Methyl-1-(4-methylphenyl)-1,2,3-triazol-4-yl]-1,2,3-triazol-4-yl]-6-(6-methoxynaphthalen-2-yl)-s-triazolo [3; **(B)** 2-methyl-5H-dibenz [b,f]azepine; **(C)** methyl 2,2-diphenyl-3-methylbutanoate; **(D)** dexamethasone (positive control).

The different phytochemicals identified from flower-derived ELNs were further subjected to molecular docking against NF-κB p65–p50 heterodimer (PDB id: 1VKX) to assess the binding affinities of the different metabolites through AutoDock Vina software. The molecular docking data were assessed according to the binding affinities (in kcal/mol) represented in Tables 8–10. The top three compounds exhibiting the highest binding scores (Table 11) along with dexamethasone were visualized for molecular interactions using PyMOL and Discovery Studio Visualizer. Key hydrogen bonds, hydrophobic interactions, and π–π stacking interactions were noted to assess the quality of binding (Figure 9). The binding affinities varied between −2.6 and −8.0 kcal/mol, indicating moderate to strong predicted binding interactions. However, it is notable that among all the tested metabolites, 7H-3-[5-methyl-1-(4-methylphenyl)-1,2,3-triazol-4-yl]-1,2,3-triazol-4-yl]-6-(6-methoxynaphthalen-2-yl)-s-triazolo [3 (Ligand A) from *N. oleander*-derived ELNs (−10.0 kcal/mol), 2-methyl-5H-dibenz [b,f]azepine (Ligand B) from *C. morifolium*-derived ELNs (−6.4 kcal/mol), and methyl 2,2-diphenyl-3-methylbutanoate (Ligand C) from *A. amica*-derived ELNs (−8.7 kcal/mol) exhibited a higher binding score with 1VKX than the positive control dexamethasone (Ligand D) (−6.9 kcal/mol). The binding conformation of the top ligands from the three flower-derived ELNs were situated within the designated active site, forming a strong interaction with the key residues (Table 8).

**TABLE 8:** Binding affinities of NELN metabolites with NF-κB p50–p65 heterodimer (PDB ID: 1VKX).

Compound	Binding energy (kcal)
7H-3-[5-Methyl-1-(4-methylphenyl)-1,2,3-triazol-4-yl]-1,2,3-triazol-4-yl]-6-(6-methoxynaphthalen-2-yl)-s-triazolo [3	−9.9
Acetamide, 2-(adamantan-1-yl)-N-(1-adamantan-1-ylethyl)	−6.3
7,9-Di-tert-butyl-1-oxaspiro (4,5)deca-6,9-diene-2,8-dione	−5.4
Diethyl phthalate	−5.2
Phenol, 2,5-bis(1,1-dimethylethyl)-	−4.8
1,3,4,5-Tetrahydroxycyclohexanecarboxylic acid	−4.8
Octadecane	−4.7
Benzoic acid, 4-ethoxy-, ethyl ester	−4.7
2-Propyl-tetrahydropyran-3-ol	−4.6
5-Nitro-2,4(1H,3H)-pyrimidinedione	−4.5
cTetradecanoic acid	−4.4
m-Xylene AND p-xylene	−4.1
Hexadecanoic acid, methyl ester	−4
Undecane	−4
Phenol	−3.9
1,3-Cyclopentanedione, 2,4-dimethyl	−3.8
n-Hexadecanoic acid	−3.7
Cyclopropane, nonyl	−3.7
3-Amino-2-oxazolidinone	−3.7
2-Oxopropanoic acid	−3.2
Pentacosane	−3.2
Silanediol, dimethyl	

**TABLE 9:** Binding affinities of CELN metabolites with NF-κB p50–p65 heterodimer (PDB ID: 1VKX).

Compound	Binding energy (kcal/mol)
2-Methyl-5H-dibenz [b,f]azepine	−7.8
Benzenamine, 4,4′-methylenebis-	−5.3
Diethyl phthalate	−5.2
2-Pyrrolidinone, 1-methyl-	−5.2
Germacrene D	−5.1
Benzothiazole, 2-(2-hydroxyethylthio)-	−4.8
Bicyclo [3.1.1]hept-2-en-6-one, 2,7,7-trimethyl-	−4.6
Methyl hexadec-9-enoate	−4.3
2-Caren-4-ol	−4.3
3,6-Octadienoic acid, 3,7-dimethyl-, methyl ester, (Z)-	−4.3
1,3-Cyclohexadiene-1-carboxaldehyde, 2,6,6-trimethyl	−4.2
Ethanol, 1-(2-butoxyethoxy)-	−4
Hexadecanoic acid, methyl ester	−4
3,4,5-Trimethylpyrazole	−4
1-Hexadecanol	−3.9
Nonanal	−3.7
n-Hexadecanoic acid	−3.7
Ethanol, 2-(2-ethoxyethoxy)-	−3.4
2-Tetradecene, (E)-	−3.3
Dimethyl sulfone	−2.7

**TABLE 10:** Binding affinities of AELN metabolites with NF-κB p50–p65 heterodimer (PDB ID: 1VKX).

Compound	Binding energy (kcal/mol)
Methyl 2,2-diphenyl-3-methylbutanoate	−8.7
N-[3-[(6-Keto-9-nitro-5H-benzo[b][1,4]benzoxazepin-7-yl)oxy]phenyl]acetamide	−7.1
2H-2a,7-Methanoazuleno [5,6-b]oxirene, octahydro-3,6,6,7a-tetramethyl	−5.5
2-Pyrrolidinone, 1-methyl	−5.2
9-Octadecenoic acid	−5
Octadecanoic acid	−4.9
cis-13-Octadecenoic acid, methyl ester	−4.7
Benzene, 1,2-dimethoxy-4-propenyl-, (Z)-	−4.6
2,3-Dihydro-3,5-dihydroxy-6-methyl-4H-pyran-4-one	−4.5
1-Hexanol, 2-ethyl	−4.1
1-[2,5-Dimethoxy-4-(methylsulfonyl)phenyl]-2-propanamine	−4.1
Ethanol, 1-(2-butoxyethoxy)-	−4
Hexadecanoic acid, methyl ester	−4
1-Hexadecanol	−3.9
Cyclopropane, nonyl	−3.7
n-Hexadecanoic acid	−3.7
Nonanal	−3.7
Ethanol, 2-(2-ethoxyethoxy)-	−3.4
Nonane	−3.4

**TABLE 11:** Top three ligands and positive control with their key interacting residues.

Compound	1VKX binding affinity (kcal/mol)	Key residue
7H-3-[5-Methyl-1-(4-methylphenyl)-1,2,3-triazol-4-yl]-1,2,3-triazol-4-yl]-6-(6-methoxynaphthalen-2-yl)-s-triazolo [3	−9.9	VAL330, PRO283, ARG277, CYS334, GLY331, ASP336
2-Methyl-5H-dibenz [b,f]azepine	−7.8	TYR444, ALA451, ASP533
Methyl 2,2-diphenyl-3-methylbutanoate	−8.7	ASP636, ARG351, ALA545, TYR538, VAL509
Dexamethasone (positive control)	−6.9	LYS188, ARG14

### 3.8 Dynamic behavior and stability evaluation of top docked complexes using molecular dynamics (MD) simulations

**FIGURE 10:**
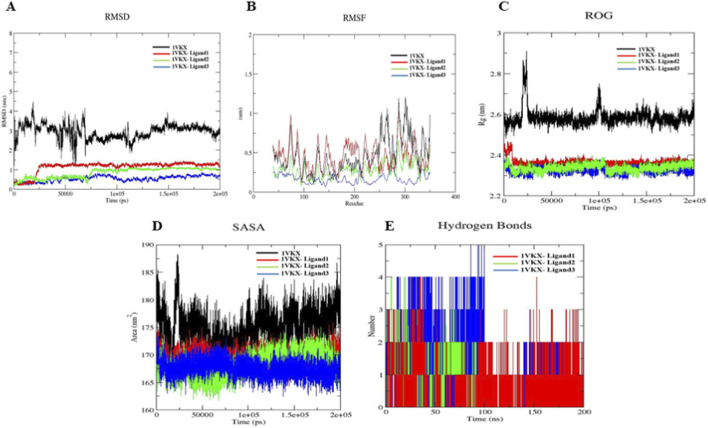
Evaluation of **(A)** RMSD, **(B)** RMSF, **(C)** ROG, **(D)** SASA, and **(E)** hydrogen bonds. Black: native protein 1VKX; red: 1VKX-Ligand 1; green: 1VKX-Ligand 2; blue: 1VKX-Ligand 3 during 200 ns simulation.

To further validate and explore the dynamic behavior of the ligand–protein interactions predicted through molecular docking, molecular dynamics (MD) simulations were performed on the top-ranked ligand–NF-κB p65–p50 heterodimer complexes. The simulations were analyzed in terms of root mean square deviation (RMSD), root mean square fluctuation (RMSF), radius of gyration (ROG), solvent accessible surface area (SASA), and the number of hydrogen bonds (H-bonds) between molecules and the protein. To evaluate the conformational stability of the protein alone and the complexes over the course of MD simulations, RMSD was monitored over a period of 200 ns (Figure 10A). The unbound protein showed significant fluctuations, with RMSD values ranging approximately 2.5–4.5 nm with occasional spikes above 6 nm, indicating substantial flexibility and conformational rearrangements. In contrast, the ligand-bound complexes exhibited improved stability throughout the simulation. It is notable from Figure 10A that the red, blue, and green lines representing different ligand–protein complexes such as 7H-3-[5-methyl-1-(4-methylphenyl)-1,2,3-triazol-4-yl]-1,2,3-triazol-4-yl]-6-(6-methoxynaphthalen-2-yl)-s-triazolo [3 (ligand 1 in red line), 2-methyl-5H-dibenz [b,f]azepine (ligand 2 in green line), and methyl 2,2-diphenyl-3-methylbutanoate (ligand 3 in blue line) stabilized within the first 20–30 ns and maintained consistent RMSD values between 0.5 and 1.5 nm. Notably, 1VKX-ligand 1 displayed the highest RMSD among the ligated systems (∼1.4 nm), while 1VKX-ligand 3 maintained the lowest RMSD (∼0.8 nm), suggesting a more stable binding conformation. To understand whether the protein was flexible or rigid, RMSF analysis was carried out. Figure 10B depicts the fluctuation of the native protein 1VKX and the other three ligand–protein complexes. The apoprotein (black line, 1VKX) demonstrated higher fluctuations throughout the sequence, particularly in the loop and terminal regions; RMSF values peaked above 1.5 nm, with the highest flexibility in the amino acid residue regions 85–110, 250–290, and 310–340. Among all the protein–ligand complexes, 1VKX-ligand 3 (blue line) showed the lowest overall RMSF values, with fluctuations mostly below 0.3 nm, indicating a strong stabilizing effect on the protein backbone. On the other hand, 1VKX-ligand 1 and 1VKX-ligand 2 exhibited intermediate stabilization, where the former complex retained slightly higher flexibility, especially around residues 90–110 and 310–330. It is also notable that residues located within the predicted ligand binding pocket (residues 135–160 and 270–290) exhibited substantially reduced fluctuations in all ligand-bound systems compared to the apoprotein. The blue complex demonstrated the greatest dampening of motion in the binding site region, suggesting that it forms the most stable interactions and may represent the most potent binder among the tested ligands. Radius of gyration (ROG) was analyzed for the apoprotein and the three complexes to understand the overall compactness and folding behavior of the protein during MD simulations (Figure 10C). The ROG of the apoprotein (black line) fluctuated between 2.55 and 2.75 nm, with intermittent spikes approaching 2.85 nm, indicating a transient conformational expansion and a relatively flexible structure. In contrast, the ligand-bound complexes maintained a more compact and stable structure throughout the 200 ns simulation time period. It is notable that the ROG values for all three complexes remained consistent in the range of 2.30 to 2.45 nm with minimal fluctuations. Among all the complexes, 1VKX-ligand 3 exhibited the lowest ROG values (∼2.32–2.36 nm), suggesting maximum structural compactness and stability upon binding. Hence, the marked difference between the apoprotein and the ligand-bound states underscores the stabilizing effect of ligand association. These results suggest that ligand binding contributes to a more ordered and compact protein structure, likely due to enhanced intramolecular interactions and reduced structural flexibility. The degree of solvent exposure of the protein in the native and ligand-bound states was analyzed using the solvent-accessible surface area (SASA) (Figure 10D). The apoprotein (1VKX, in black) exhibited higher SASA values of 170–185 nm^2^, indicating a more solvent-exposed and less compact structure. However, in comparing the SASA values, the ligand-bound complexes demonstrated reduced SASA values, 1VKX-ligand 3 (blue) maintained the lowest solvent accession (∼162–167 nm^2^), and the other two complexes (in green and red) showed moderate solvent accession (165–171 nm^2^) throughout the simulation. These findings support the observation that ligand binding reduces solvent accessibility by promoting protein compaction, in line with ROG and RMSF data. Notably, the blue complex again demonstrated the highest structural integrity and least solvent-exposed conformation. Subsequently, hydrogen bonding (H-bond) analysis was performed to monitor the stability and persistence of the binding interactions over the simulation period (Figure 10E). Consistently, 1VKX-ligand 3 and 1VKX-ligand 2 exhibited 5–7 and 2–4 instances of H-bonds, respectively, during most of the simulation time, while 1VKX-ligand exhibited 1–2 H-bonds, which were less persistent and stable over the simulation time. The H-bond analysis data for 1VKX-ligand 3 accords with the RMSD, RMSF, ROG, and SASA analyses. Hence, the trajectory analysis of the MD simulation revealed enhanced stability, compactness, and reduced flexibility in the ligand-bound complexes, with ligand 3 (methyl 2,2-diphenyl-3-methylbutanoate) exhibiting the most stable binding interaction with 1VKX.

### 3.9 Analysis of binding free energies for the topmost interactions

**FIGURE 11:**
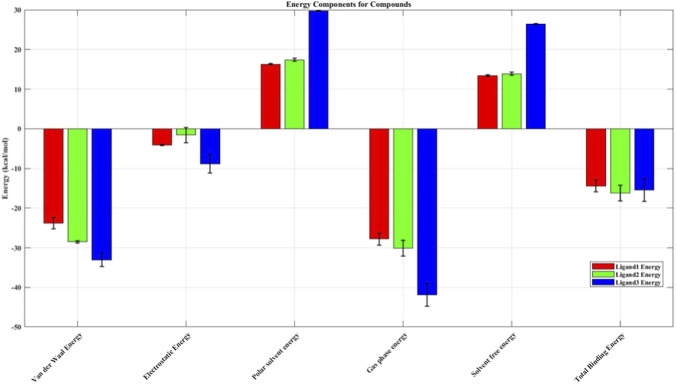
Computed binding free energies for Complexes 1, 2, and 3 using MMPBSA.

MMPBSA calculations were used to determine the binding free energies—the energy change between bound and unbound states of the protein and ligand (Figure 11) for Complexes 1, 2, and 3. Table 12 demonstrates the total binding free energy as −14.42 kcal/mol for Complex 1, –15.48 kcal/mol for Complex 2, and –16.25 for Complex 3. It is notable from Figure 11 and Table 12 that Complex 3 (methyl 2,2-diphenyl-3-methylbutanoate – 1VKX) exhibited the least total binding free energy, demonstrating the ligand’s strong interactions with 1VKX.

**TABLE 12:** Computed binding free energies for the complexes using MMPBSA.

S. no.	Energy component (kcal/mol)	Complex 1		Complex 2		Complex 3	
		Average	S.D	Average	S.D	Average	S.D
1	Van der Waal energy	−23.80	1.43	−33.04	1.70	−28.50	0.24
2	Electrostatic energy	−4.05	0.11	−8.88	2.33	−1.62	1.90
3	Polar solvent energy	16.33	0.21	29.70	0.12	17.43	0.38
4	Gas-phase energy	−27.85	1.44	−41.91	2.89	−30.12	1.97
5	Solvent free energy	13.44	0.21	26.43	0.12	13.87	0.39
6	Total binding free energy	−14.42	1.45	−15.48	2.89	−16.25	2.01

## 4 Discussion

Flowers are rich reservoirs of diverse phytochemicals and have been used traditionally worldwide. However, recent focus has been shifting to studying flower-derived ELNs owing to their potential therapeutic attributes, enhanced biocompatibility, and minimal toxicity. In the present study, the integration of metabolomics with *in vitro* and computational analyses allowed us to examine how floral ELNs derived from three different types of flower may influence inflammatory pathways, particularly NF-κB signaling. Physiological characterization of the isolated ELNs through nanoparticle tracking assay confirmed the presence of nanovesicles with a size distribution consistent with that of exosomes, ranging 100–200 nm. Additionally, zeta potential was measured to evaluate the surface charge of ELNs, their stability, reduced aggregation in solution, and their ability to be absorbed by recipient cells (Emmanuela et al., 2024). The flower-derived ELNs exhibited a zeta potential of approximately −10 mV to −40 mV. These properties indicate that the isolated ELNs from different flowers support the size and stability of their potential bioavailability as a drug delivery system.

Flower-derived ELNs are also known to encapsulate different bioactive compounds involved in mediating inter-species signal transduction and have significant therapeutic potential against various diseases (Liu et al., 2022). The GC-MS chromatogram obtained in our present study identified several encapsulated metabolites within the flower-derived ELNs, many of which have been previously reported in the literature as possessing therapeutic properties. These findings support the growing concept that plant ELNs naturally package functionally enriched metabolites capable of inter-species signaling, thus suggesting their potential therapeutic relevance. The GC-MS chromatogram in our present study highlighted the presence of different potential bioactive compounds encapsulated in the flower-derived ELNs which are helpful in treating inflammatory conditions and oxidative stress and have antibacterial, antiparasitic, and antitumor properties such as hexadecanoic acid, methyl ester, benzoic acid, 4-ethoxy-, ethyl ester (NELNs), germacrene D, 2-Caren-4-ol (CELNs), 9-octadecenoic acid, and n-hexadecanoic acid (AELNs) (Sheela and Uthayakumari, 2013; Ukwubile et al., 2019; Montanari et al., 2011; Salah et al., 2024; Ganesan et al., 2024; Sympli, 2021). However, few of the phytocompounds, such as N-[3-[(6-keto-9-nitro-5H-benzo[b][1,4]benzoxazepin-7-yl)oxy]phenyl]acetamide, 7H-3-[5-methyl-1-(4-methylphenyl)-1,2,3-triazol-4-yl]-1,2,3-triazol-4-yl]-6-(6-methoxynaphthalen-2-yl)-s-triazolo [3, and methyl 2,2-diphenyl-3-methylbutanoate, identified in the flower-derived ELNs have been reported in the literature, suggesting that floral vesicles may harbor a broader metabolite spectrum than currently recognized.

Building on the chemical composition of floral ELNs revealed by GC-MS analysis, we subsequently evaluated free radical scavenging activity. The identification of multiple antioxidant-associated metabolites through GC-MS likely contributed to the dose-dependent free radical scavenging activity displayed by the ELNs, supporting a functional link between their chemical composition and biological behavior. These findings align with previous studies showing that plant-derived ELNs can retain and deliver bioactive molecules capable of neutralizing oxidative stress (Perut et al., 2021; Jun et al., 2021). The observed antioxidant activity suggests that flower-derived ELNs can help mitigate oxidative damage, further supporting their potential as therapeutic agents in inflammation-related disorders where oxidative stress plays a critical role.

To understand the therapeutic attributes of the flower-derived ELNs *in vitro*, RAW264.7 macrophages were treated with different doses of NELNs, CELNs, and AELNs. Minimal toxicity was observed at lower dosages, whereas higher doses exhibited significant toxicity (Figure 6). This remains consistent with previous findings on ELNs isolated from edible tea flowers, *Catharanthus roseus* (Ou et al., 2023; Chen et al., 2022), which also showed minimal toxicity in *in vitro* studies. Subsequently, to validate the effect of floral ELNs on NF-κB activity, TNF-α and IL-10 mRNA expression, LPS-induced RAW264.7 macrophages were treated with the flower-derived ELNs in selected doses based on our MTT data. Notably, the ELN-treated groups showed a significant reduction in NF-κB DNA binding activity compared to the LPS group, suggesting a possible modulatory effect of floral ELNs on NF-κB signaling. Moreover, NF-κB is a crucial signaling cascade activated by several inflammatory mediators; upon activation, it leads to the transcription of different pro-inflammatory cytokines, thus elevating inflammatory conditions (Leclercq et al., 2025). TNF-α is a pro-inflammatory cytokine which plays a crucial role in immune response and regulation of inflammation (van Loo and Bertrand, 2022). LPS treatment of RAW macrophages is known to elevate the levels of pro-inflammatory cytokines (TNF-α) (Anilkumar et al., 2017). Gene expression studies have revealed an elevated TNF-α mRNA expression in LPS treatment, which was further ameliorated by ELN treatment. There was also a decline in IL-10 expression in the LPS-treated condition, which was further reversed back in ELN treated groups. IL-10 is an anti-inflammatory cytokine which inhibits the production of several pro-inflammatory mediators, including TNF-α (Murr, 2005).

To evaluate the pharmacokinetic and safety parameters of metabolites from the flower-derived ELNs, Lipinski’s rule of five and the ProTox prediction server were used. Most of the identified phytochemicals complied with Lipinski’s rule of five, suggesting good potential for oral bioavailability (Rai et al., 2023). Notably, several aliphatic esters and alcohols such as hexadecanoic acid methyl ester, nonanal, and dimethyl sulfone, as well as cyclic and aromatic compounds like phenol derivatives, demonstrated physicochemical properties within the accepted thresholds. Minor deviations in logP or molecular weight observed in compounds like pentacosane or nonadecane derivatives are not uncommon in lipid-soluble phytochemicals and may not necessarily preclude drug-likeness, particularly when encapsulated in ELNs that can enhance solubility and membrane transport (Kumari et al., 2014). This highlights the advantage of ELN encapsulation as a delivery mechanism that could overcome classic drug-likeness limitations, especially for hydrophobic natural compounds (Kumari et al., 2014). ProTox-II analysis revealed low predicted systemic toxicity for most of the compounds, with LD_50_ values largely ranging between 500 and 10,000 mg/kg, placing them within toxicity classes 3 to 6. A substantial number of metabolites from all three flower sources were predicted to be non-hepatotoxic, non-neurotoxic, and non-nephrotoxic, indicating a generally favorable safety profile. While a few compounds such as 3-amino-2-oxazolidinone and phenol showed moderate predicted organ toxicity or lower LD_50_ values, their toxic effects are likely to be dose-dependent and may be mitigated by ELN-mediated delivery, which can limit systemic exposure and enhance tissue targeting (Rai et al., 2023). Furthermore, high-molecular-weight compounds with lipid-like structures, which might otherwise accumulate in tissues if administered freely, may benefit from controlled release and clearance profiles when encapsulated in ELNs. Across the three floral species, a convergence in safety and drug-likeness trends was observed. For instance, metabolites such as dimethyl sulfone, n-hexadecanoic acid, and 1-hexadecanol were consistently present with favorable pharmacokinetic properties and minimal toxicity across *Nerium oleander*, *Chrysanthemum morifolium*, and *Agave amica* ELNs. This convergence in safety and drug-likeness trends supports the hypothesis that floral ELNs serve as naturally optimized nanocarriers, selectively enriching bioactive compounds with therapeutic promise.

Moreover, mechanistic insights from molecular docking and molecular dynamics (MD) simulations further strengthen the therapeutic relevance of the identified ELN metabolites.

Molecular docking studies revealed that several phytocompounds identified from *N. oleander*, *C. morifolium*, and *A. amica* ELNs exhibited moderate to strong binding affinities with 1VKX, a key transcriptional regulator in inflammation. Notably, metabolites such as 7H-3-[5-methyl-1-(4-methylphenyl)-1,2,3-triazol-4-yl]-1,2,3-triazol-4-yl]-6-(6-methoxynaphthalen-2-yl)-s-triazolo[3 from *N. oleander* (−10.0 kcal/mol), 2-methyl-5H-dibenz [b,f]azepine from *C. morifolium* (−7.8 kcal/mol), and methyl 2,2-diphenyl-3-methylbutanoate from *A. amica* (−8.7 kcal/mol) demonstrated stronger binding affinities than the reference anti-inflammatory drug dexamethasone (−6.9 kcal/mol). Interaction studies revealed the key residues involved in the top interactions (Table 10). Positively charged residues such as Arg277, Arg351, and Lys188 substantially contribute to DNA binding and the stability of the NF-κB–DNA complex. In addition, hydrophobic residues including Val330, Pro283, Ala545, and Val509 contribute to structural stability and ligand interaction within the NF-κB binding pocket, potentially influencing conformational dynamics (Chen et al., 1998; Hayden and Ghosh, 2008). Notably, acidic residues such as Asp336, Asp533, and Asp636 are implicated in protein–protein interactions and regulatory conformational control of NF-κB signaling (Li et al., 2025). These findings suggest that specific ELN-associated compounds may serve as effective modulators of NF-κB signaling. To further substantiate these static docking interactions, MD simulations were employed to assess the stability, conformational dynamics, and persistence of binding under near-physiological conditions. Unlike docking, which represents a single static pose, MD captures the temporal evolution of protein–ligand interactions, providing critical insight into how stable and biologically relevant these complexes are over time (Subramani and Venugopal, 2025). All three ligand–protein complexes exhibited reduced RMSD and RMSF values compared to the unbound protein, indicating greater structural stability and reduced flexibility. In particular, the 1VKX–ligand 3 complex (methyl 2,2-diphenyl-3-methylbutanoate) showed the lowest RMSD (∼0.8 nm), lowest radius of gyration (∼2.32–2.36 nm), and minimal solvent-accessible surface area (SASA), suggesting the formation of a highly compact and stable structure upon ligand association. These parameters collectively indicate that ligand binding restricts backbone flexibility and promotes a more compact conformation, potentially limiting the structural rearrangements necessary for NF-κB dimer activation and DNA binding. Moreover, persistent hydrogen bonding (5–7 interactions) throughout the simulation further validated the strong and stable interaction of this compound with the protein’s active site. These findings collectively highlight the structure-stabilizing and potentially inhibitory role of flower-derived ELN metabolites on NF-κB p65–p50 complex activity. The ability of ELN-encapsulated phytocompounds to not only engage in strong binding but also reinforce protein rigidity suggests that ELNs offer a dual advantage: delivering bioactive molecules efficiently and enhancing their therapeutic efficacy through stable, targeted molecular interactions. Among the tested compounds, the AELN-derived metabolite methyl 2,2-diphenyl-3-methylbutanoate emerged as the most promising lead candidate, exhibiting superior docking and simulation performance across all stability metrics. Furthermore, the MMPBSA results align strongly with these dynamic signatures. The most favorable binding free energies were predicted for complex 3 (methyl 2,2-diphenyl-3-methylbutanoate – 1VKX) (−16.25 kcal/mol), consistent with their persistent interactions, stable conformational ensembles, and well-defined free-energy basins. These affinities fall within ranges observed for bioactive plant-derived small molecules reported as modulating inflammation associated pathways. Weaker binding for Complex 1 interaction, while still energetically favorable, correlates with their comparatively higher RMSD fluctuations and broader conformational sampling, indicating more flexible but viable engagement modes.

Together, these results underscore the potential of floral ELNs as a nanocarrier system for anti-inflammatory phytochemicals, particularly in targeting NF-κB-driven pathologies. This study adds to the growing body of evidence that plant-derived ELNs can serve as a cell-free, biocompatible therapeutic platform with enhanced molecular engagement, offering a new avenue for natural product-based drug development.

## 5 Conclusion

The findings of the present study suggest the potential of flower-derived ELNs in offering new avenues in therapeutic interventions and novel drug discovery. Our results highlight the potential future application of using plant-derived ELNs in therapeutic intervention for the pharmaceutical industry, which could help with dose optimization and toxicity-related issues. Opting for natural ELN sources draws on native therapeutic ingredients which could even substantiate the futuristic application of ELNs in various applications. The present study highlights the potential of the floral extracellular vesicles to modulate the mRNA expression of key inflammatory markers such as TNF-α and IL-10 and regulate the NF-κB transcriptional activity. Furthermore, the identified ELN-associated metabolites showed favorable drug-likeness and low predicted toxicity, and docking and molecular dynamics simulations suggested plausible interaction patterns with the NF-κB p65–p50 complex. While the current data highlight the potential utility of floral ELNs as natural, biocompatible carriers, further mechanistic studies and *in vivo* validation are necessary to establish their pharmacological action. Overall, this comprehensive *in silico* and *in vitro* assessment on floral ELNs offers an initial foundation for further pharmacological investigations and contributes to the growing interest in using exosome-like nanoparticles as safe and efficient natural drug delivery systems.

## Data Availability

The raw data supporting the conclusions of this article will be made available by the authors, without undue reservation.
