# Supplementary figures and images for: Integrated experimental and computational insights into the anti-inflammatory potential of flower-derived exosome-like nanoparticles targeting the NF-κB pathway
Source: Front Bioinform. 2026 Feb 16;6:1737325. doi: 10.3389/fbinf.2026.1737325 (PMC12950680; doi:10.3389/fbinf.2026.1737325)

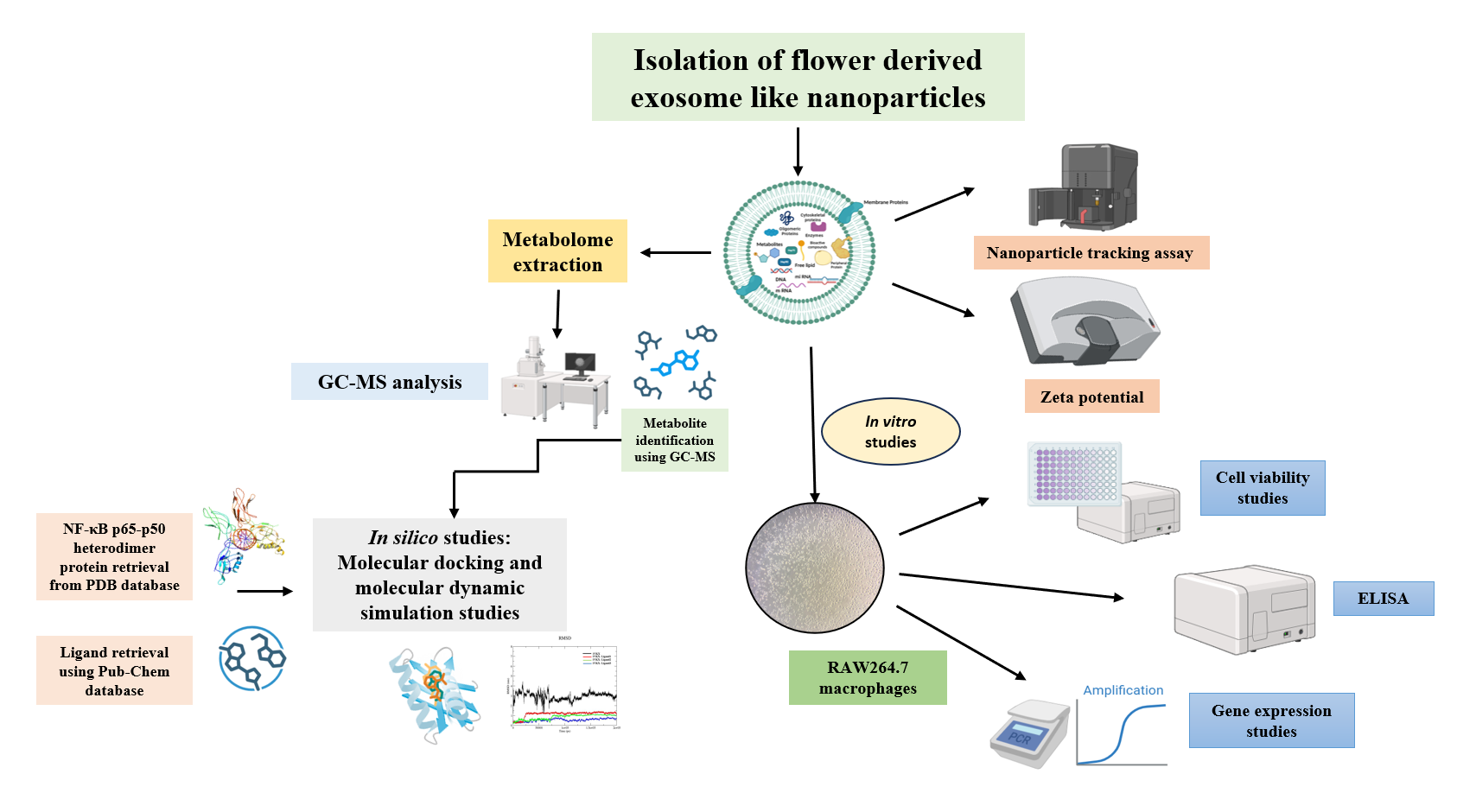

Supplement: Supplementary file 1 [file Image1.png]
